# Supplementary material for: Clinicopathological, endocrine, and oxidative stress alterations in dairy cattle naturally affected by bovine ephemeral fever
Source: Ir Vet J. 2026 May 25;79:29. doi: 10.1186/s13620-026-00347-1 (PMC13220354; doi:10.1186/s13620-026-00347-1)
Supplement: Supplementary file 1 — Supplementary Material 1. [file 13620_2026_347_MOESM1_ESM.pdf]

Original full blot for Figure 1

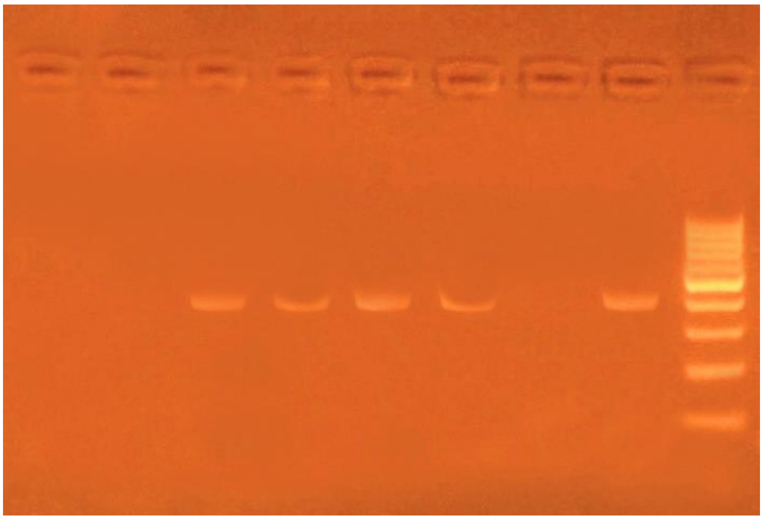

FIGURE 1 Agarose gel electrophoresis of RT-PCR products of G gene region of BEFV

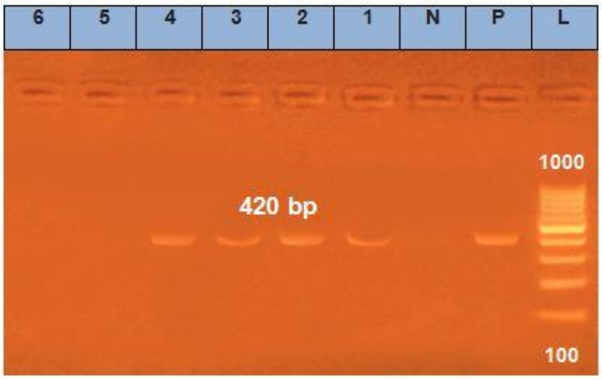

FIGURE 1 Electrophoretic pattern of the amplified products 420 bp of the G gene for BEF virus from blood samples of the infected cattle by RT-PCR. P: Reference BEF virus (Control positive), L: Nucleic acid marker (100bp- 1000bp), Lanes 1 - 6: Buffy coat samples from suspected cattle. Lanes 1, 2. 3and 4 were positive samples.
